# Telemedicine follow-up and nutritional outcomes in children with neurological impairment: a longitudinal study

**DOI:** 10.3389/fped.2026.1868448

**Published:** 2026-07-08

**Authors:** Francesca Eletti, Veronica Perico, Alessandro Visioli, Chiara Montanari, Veronica Maria Tagi, Sara Vizzuso, Valeria Calcaterra, Barbara Borsani, Gianvincenzo Zuccotti

**Affiliations:** 1 Department of Biomedical and Clinical Sciences, University of Milan, Milan, Italy; 2 Department of Pediatrics, Vittore Buzzi Children’s Hospital, Milan, Italy; 3 Metabolic Diseases Unit, Department of Pediatrics, Vittore Buzzi Children’s Hospital, Milan, Italy; 4 Pediatric and Adolescent Unit, Department of Internal Medicine, University of Pavia, Pavia, Italy

**Keywords:** gastrostomy, GMFCS, malnutrition, neurological impairment, nutritional status, pediatrics, telemedicine

## Abstract

**Background:**

Children with neurological impairment are at high risk of malnutrition and require regular nutritional monitoring. Telemedicine has been increasingly used to support follow-up visits, but its impact on nutritional outcomes remains unclear.

**Methods:**

We conducted a longitudinal observational study including 152 children with neurological impairment. Patients received either standard in-person follow-up or a combination of in-person and telemedicine visits. Nutritional status was assessed using body mass index (BMI) z-score at baseline and follow-up. Nutritional outcome was classified as improved, stable, or worsened based on changes in BMI z-score. The aim of this study was to evaluate the association between telemedicine follow-up and nutritional outcomes and to identify clinical factors associated with nutritional worsening. Multivariable logistic regression was performed to identify predictors of nutritional deterioration.

**Results:**

At baseline, children in the telemedicine group were younger and had a higher prevalence of percutaneous endoscopic gastrostomy (PEG) (*p* = 0.013), while no differences were observed in gross motor function classification system (GMFCS) severity or baseline nutritional status. No significant differences were observed between groups in BMI z-score at follow-up (*p* = 0.877), change in BMI z-score (*p* = 0.458), nutritional status at follow-up (*p* = 0.356), or nutritional outcome (*p* = 0.329). In multivariable analysis, baseline BMI z-score was associated with nutritional worsening (OR 1.23, 95% CI 1.04-1.50; *p* = 0.013), whereas telemedicine follow-up (*p* = 0.166), age (*p* = 0.767), PEG (*p* = 0.869), GMFCS severity (*p* = 0.704), and follow-up duration (*p* = 0.352) were not associated with worsening.

**Conclusion:**

Baseline BMI z-score was the only variable independently associated with nutritional worsening during follow-up. No statistically significant differences in nutritional outcomes were observed between telemedicine and standard care groups; however, differences in baseline characteristics limit direct comparability. Telemedicine may represent a feasible and complementary approach to support follow-up in nutritionally vulnerable pediatric patients.

## 1 Introduction

Telemedicine (TLM) has become an established component of pediatric healthcare and is increasingly integrated into routine clinical practice worldwide. Telemedicine has been increasingly adopted in pediatric care because it can improve access to specialty services while reducing the travel-related burden for families, although implementation barriers such as workflow integration, technology reliability, and reimbursement remain relevant (1, 2). In recent years, the rapid evolution of digital health technologies has enabled the development of new models of care delivery, supporting remote monitoring, clinical decision-making, and patient-provider communication. Its implementation has been further accelerated by the COVID-19 pandemic, leading to the development of structured digital care pathways and the formal recognition of telemedicine within national healthcare systems, including the Italian National Health System, following guidelines issued in 2020 (3–6). Regional pediatric telemedicine models have demonstrated the potential to improve quality of care, optimize resource allocation, and ensure continuity of healthcare delivery through remote services (3, 7). In pediatric neurology, telemedicine has shown particular relevance in supporting long-term management of children with chronic and complex conditions. These patients often require frequent multidisciplinary follow-up in tertiary care centers, placing a substantial burden on families in terms of travel, time, and organizational demands. Large pediatric neurology telehealth cohorts have shown that telemedicine is feasible for a substantial proportion of outpatient encounters and can support continuity of care without compromising routine follow-up (8). Telemedicine has been shown to reduce this burden while maintaining continuity of care and facilitating communication between healthcare providers and caregivers (4, 5, 9–11). Moreover, telemedicine may facilitate more frequent clinical interactions, potentially allowing for earlier identification of clinical changes and more timely interventions. In addition, digital health technologies can reduce geographical barriers and waiting times, improving access to specialized care and enabling more continuous and personalized management, particularly in children with rare neurological diseases (5, 10, 12). Children with neurological impairment represent a highly vulnerable population from a nutritional perspective. Children with neurological impairment are at high risk of malnutrition, and the severity of nutritional problems increases with the degree of neurological impairment and the presence of oromotor dysfunction (13, 14). Feeding difficulties, growth impairment, oropharyngeal dysphagia, gastrointestinal disorders, and increased metabolic demands contribute to a high prevalence of malnutrition in this population (15–17). Malnutrition has been consistently associated with worse clinical outcomes, increased morbidity, reduced quality of life, increased hospitalization rates and reduced functional outcomes (18–21). Furthermore, inadequate nutritional status may negatively impact growth, immune function, and overall disease progression, further increasing the complexity of clinical management. Nutritional management therefore represents a key component of care and requires regular and structured monitoring, including anthropometric assessment, dietary evaluation, and, when necessary, enteral nutrition support (18, 21, 22). This process is typically embedded within a multidisciplinary framework involving pediatricians, neurologists, nutritionists and rehabilitation specialists. Despite the need for frequent monitoring, regular in-person visits may be challenging for these patients due to mobility limitations, medical complexity, and the logistical burden on families. In addition, healthcare access may be further limited in geographically remote or resource-constrained settings, where specialized services are not readily available. In this context, telemedicine has been proposed as a promising strategy to support clinical and nutritional follow-up while reducing barriers to care (9, 10, 23). However, these studies have primarily evaluated process-related outcomes rather than objective clinical endpoints. Despite these advances, evidence regarding the impact of telemedicine on clinical outcomes remains limited (24, 25), particularly in children with severe neurological impairment. In particular, few studies have specifically evaluated nutritional outcomes, despite their central role in this population. Moreover, existing evidence is often heterogeneous, based on small samples, or focused on short-term outcomes, highlighting the need for further research in real-world clinical settings (10, 22, 23). This gap is particularly relevant given the increasing integration of telemedicine into routine care pathways. Maintaining nutritional stability is therefore a clinically meaningful outcome in this population. In children with neurological impairment, the goal of nutritional follow-up is not only to improve nutritional status, but also to prevent deterioration over time, which may occur due to disease progression or feeding difficulties. In this context, telemedicine should be considered as a tool to support follow-up rather than a direct determinant of clinical outcomes. Understanding whether telemedicine-based follow-up is associated with stable or improved nutritional outcomes, without adversely affecting clinical trajectories, is therefore of clinical relevance. The aim of this study was to evaluate the association between telemedicine-based follow-up and nutritional outcomes in a cohort of children with neurological impairment in a real-world clinical setting, and to identify independent clinical factors associated with nutritional worsening.

## 2 Materials and methods

We conducted a longitudinal observational study including children with neurological impairment followed at a tertiary pediatric referral center, “*Vittore Buzzi” Children's Hospital*, Milan, Italy between 2024 and 2025. All patients attending the outpatient clinic for nutritional evaluation during the study period were screened for eligibility. Children and adolescents (aged 0-18 years) were included if they had a confirmed diagnosis of neurological impairment and at least two nutritional assessments, consisting of baseline in-person visit and at least one follow-up evaluation. Follow-up assessments could be performed either in person or via telemedicine, according to clinical practice. Anthropometric measurements were collected during in-person visits, while during telemedicine follow-up they were reported by caregivers as described below. Follow-up visits were scheduled according to clinical practice, typically between 3 and 12 months from baseline. Patients were excluded from analyses involving nutritional outcomes if baseline anthropometric data were missing or if follow-up data were not available. All patients underwent an initial in-person visit. Based on clinical needs and organizational factors, follow-up was performed either through standard in-person visits only or through a combination of in-person and telemedicine visits. Patients were therefore categorized into two groups: standard care (in-person visits only) and telemedicine follow-up (combined in-person and remote visits).

### 2.1 Data collection

Clinical and demographic data were collected during routine clinical visits and recorded in the electronic medical records. Demographic variables included age and sex. Clinical variables included underlying diagnosis, Gross Motor Function Classification System (GMFCS), and presence of percutaneous endoscopic gastrostomy (PEG) (yes/no). Anthropometric data included weight and height/length, from which BMI and BMI z-scores were calculated at baseline and follow-up, as described below. Follow-up variables included duration of follow-up (months) and type of follow-up (standard care vs telemedicine).

The diagnoses were grouped into the following categories: encephalopathies (including cerebral palsy), genetic syndromes, neuromuscular diseases, neurodegenerative diseases, and neurometabolic diseases.

For statistical analysis, GMFCS levels (26–28) were dichotomized into non-severe (levels I-III) and severe (levels IV-V), in line with commonly used classifications distinguishing ambulatory from non-ambulatory patients.

### 2.2 Anthropometric measurements and nutritional assessment

Anthropometric measurements were primarily collected during in-person visits by trained healthcare professionals according to standard clinical procedures. Body weight was measured using calibrated digital scales. For children unable to stand independently, weight was obtained using alternative methods (e.g., wheelchair scales or caregiver-assisted weighing), according to clinical practice. Height or length was measured using a stadiometer in children able to stand. In children with severe motor impairment who were unable to stand, estimated height was obtained using segmental measurements (e.g., knee height) and validated predictive equations when appropriate (29). In patients receiving telemedicine follow-up, when in-person measurements were not available, anthropometric data were reported by caregivers, who had been previously instructed on standardized measurement procedures. Before telemedicine follow-up, caregivers received verbal instruction during in-person visits on standardized anthropometric measurement procedures. For body weight assessment, caregivers were trained to use a caregiver-assisted double-weighing method, consisting of weighing the caregiver while holding the child and subsequently weighing the caregivers alone. For children unable to stand independently, caregivers were also instructed on how to obtain knee-heel length measurements, which were then used by healthcare professionals to estimate stature using validated predictive equations. Body weight was estimated using caregiver-assisted double weighing, while height was estimated using knee-heel length and predictive equations, as described above. These measurements were used for clinical monitoring between in-person visits and were interpreted with caution. BMI was calculated as weight (kg) divided by height squared (m^2^). BMI z-scores used for statistical analyses were derived using World Health Organization (WHO) growth standards for children younger than 2 years of age (30) and Centers for Disease-Control and Prevention (CDC) for children aged 2 years and older. Disease-specific growth charts, when available (e.g., cerebral palsy, spinal muscular atrophy, Rett syndrome, and Prader-Willi syndrome), were used to support clinical nutritional assessment and percentile interpretation but were not used to derive the BMI z-score included in the statistical analyses (31–35). BMI was used as the primary indicator of nutritional status across the cohort to ensure consistency of assessment, although its interpretation in children under 2 years of age was considered with caution. BMI z-scores are widely used to assess and monitor growth trajectories in pediatric populations (36). Nutritional status at baseline was classified according to established WHO definitions (30):

- Severe malnutrition (BMI z-score < −3)
- Moderate malnutrition (−3 ≤ BMI z-score < −2)
- At risk of malnutrition (−2 ≤ BMI z-score < −1)
- Normal nutritional status (−1 ≤ BMI z-score ≤ 1)
- Overweight (1 < BMI z-score ≤ 2)
- Obesity (BMI z-score > 2)

Change in nutritional status was assessed as the difference between BMI z-score at follow-up and baseline BMI z-score (Δ BMI z-score), using the first available follow-up measurement after baseline, as commonly applied in longitudinal pediatric studies (37). To ensure comparability across participants, analyses were restricted to the first available follow-up visit because the number and timing of subsequent follow-up assessments varied substantially among patients as part of ongoing routine clinical care.

Changes in BMI z-score (Δ BMI z-score) were used to assess nutritional trajectories over time. Nutritional outcome was categorized based on changes in BMI z-score, using a threshold of ± 0.5 to define clinically relevant variation. This threshold was selected to account for measurement variability and to distinguish true changes in nutritional status from minor fluctuations commonly observed in clinical practice. A difference of 0.5 standard deviation scores has been proposed as a benchmark for clinically meaningful differences in pediatric growth assessment (38–40). Given the lack of universally accepted cut-offs for clinically meaningful longitudinal changes in BMI z-score among children with neurological impairment, this threshold was adopted as a pragmatic criterion.

- Improved (Δ BMI z-score > + 0.5)
- Stable (−0.5 ≤ Δ BMI z-score ≤  + 0.5)
- Worsened (Δ BMI z-score < −0.5)

For regression analysis, nutritional outcome was dichotomized into worsened versus stable/improved.

Follow-up duration was defined as the time between baseline and the first available follow-up visit and ranged from 3 to 12 months. Due to the observational nature of the study, follow-up intervals were not standardized and reflected routine clinical practice.

### 2.3 Telemedicine follow-up

Telemedicine visits were conducted using a dedicated digital platform (COD20) (7). These visits included clinical assessment based on caregiver-reported information, such as feeding tolerance, gastrointestinal symptoms, and overall clinical condition. Nutritional assessment during telemedicine visits was based on the review of recent anthropometric data, when available, dietary intake, and feeding practice, as reported by caregivers. Anthropometric measurements, when collected remotely, were obtained through caregiver-assisted measurements. Body weight was measured using a caregiver-assisted weighing method, while height was estimated using segmental measures (e.g., knee height) according to validated methods (29). Caregivers were instructed on measurement procedures. Telemedicine visits also included dietary counseling and structured caregiver interviews aimed at monitoring nutritional status, identifying potential feeding difficulties, and providing tailored nutritional recommendations. Telemedicine follow-up was integrated with standard in-person care and was primarily used for patients requiring closer monitoring or presenting with higher clinical complexity. Telemedicine program was implemented to increase the frequency of nutritional monitoring and reduce delays between follow-up assessments in a clinically fragile population. According to the clinical protocol, patients were generally scheduled for an in-person baseline evaluation followed by telemedicine follow-up visits at approximately 2 and 4 months, and an in-person reassessment at approximately 6 months. However, because the study is ongoing and follow-up schedules reflect routine clinical practice, not all participants had completed the same sequence of visits at the time of analyses. Patients were classified in the telemedicine group if they received at least one telemedicine follow-up visit during the study period.

### 2.4 Statistical analysis

Continuous variables are presented as mean ± standard deviation, and categorical variables as counts and percentage. Baseline characteristics between groups were compared using t-test for continuous variables and chi-square or Fisher's exact test for categorical variables, as appropriate. Change in BMI z-score was calculated at the individual level as the difference between follow-up and baseline values (Δ BMI z-score). Between-group comparisons of BMI z-score at follow-up and Δ BMI z-score were performed using t-tests, while categorical outcomes were compared using chi-square or Fisher's exact tests, as appropriate. A multivariable logistic regression analysis was performed to identify factors associated with nutritional worsening. Variables included in the multivariable model were selected based on clinical relevance and prior evidence. The dependent variable was nutritional worsening (yes/no). Independent variables included telemedicine follow-up, age, PEG, GMFCS severity, baseline BMI z-score, and follow-up duration. Baseline clinical variables (such as PEG and GMFCS) were included as indicators of disease severity and potential confounders. As a sensitivity analysis, propensity score matching (PSM) was performed to reduce baseline differences between patients receiving telemedicine and those receiving standard care. Propensity scores were estimated using age, sex, PEG status, GMFCS severity, diagnostic category, and baseline BMI z-score. One-to-one nearest-neighbor matching without replacement was applied using a caliper of 0.2. Baseline covariate balance after matching was assessed using standardized mean differences. Statistical significance was set at *p* < 0.05. Primary analyses were performed using JMP software. Propensity score matching was conducted in R (version 4.6.0). No imputation was performed for missing data.

Analyses did not explicitly account for the paired nature of repeated measurements, which may have limited the ability to detect within-subject changes over time, given the variability in follow-up timing and the observational design.

### 2.5 Ethics statement

This study was approved by the local Ethics Committee (Lombardia 1, protocol number 81-2024) and conducted in accordance with the principles of the Declaration of Helsinki. Written informed consent was obtained from the parents or legal guardians of all participating children after full information on the nature of the study had been provided.

## 3 Results

**Figure 1:**
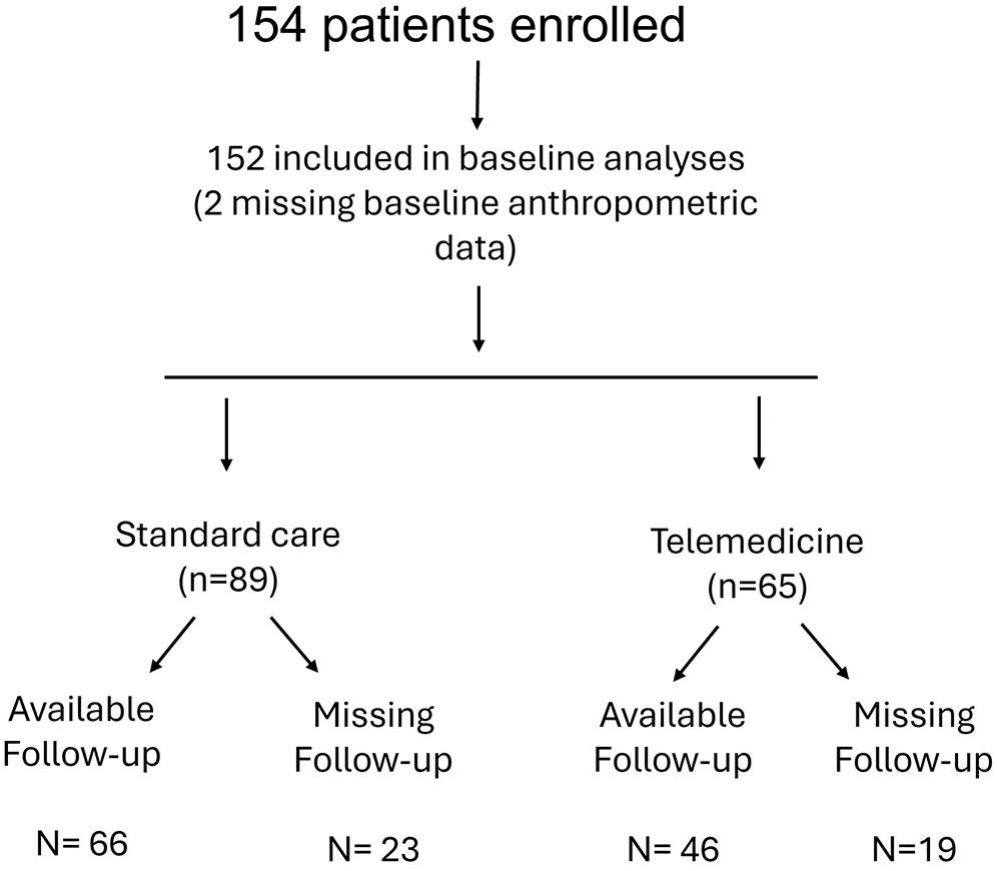
Study flowchart. Flowchart showing participant inclusion, availability of baseline nutritional data, allocation to follow-up modality, and follow-up completion.

A total of 154 children with neurological impairment were included in the study (Figure 1).

The mean age was 8.52 ± 4.85 years (range: 9 months to 18 years), and 97 (63%) were male. Of these, 65 (42.2%) received telemedicine follow-up, while 89 (57.8%) received standard in-person care only. Among patients in the telemedicine group, the median number of telemedicine visits completed during the study period was 2 (IQR 2-3). The clinical and nutritional characteristics of the overall study population are reported in Supplementary Table 1.

The prevalence of malnutrition at baseline was high in the overall population (Table 1). Baseline nutritional data were available for 152 patients. Severe malnutrition was present in 32.9% of children and moderate malnutrition in 19.1%, with a mean BMI z-score at baseline of −2.30 ± 2.87. Malnutrition at baseline was significantly associated with the underlying diagnosis (*p* = 0.001) and the presence of PEG (*p* = 0.049), while no significant association was found with age, sex, or telemedicine group (Table 2). Baseline characteristics according to telemedicine exposure are reported in Table 2. Children in the telemedicine group were younger compared to those receiving standard care; however, this difference was not statistically significant (7.6 ± 4.6 vs 9.1 ± 4.8 years, *p* = 0.056). No significant differences were observed in baseline BMI z-score (*p* = 0.158) or in the distribution of nutritional status categories (*p* = 0.430). The prevalence of PEG was significantly higher in the telemedicine group (58.5% vs 38.2%, *p* = 0.013). No differences were observed in GMFCS severity between groups (*p* = 0.852). The distribution of underlying diagnoses differed significantly between groups (*p* = 0.035), with a higher proportion of genetic syndromes in the telemedicine group, while encephalopathies remained the most represented category in both groups.

**Table 1:** Nutritional status at baseline.

Nutritional status	*N*	%
Severe malnutrition	50	32.9
Moderate malnutrition	29	19.1
Normal	57	37.5
Overweight	10	6.6
Obesity	6	3.9
Total	152^a^	100

Mean BMI z-score at baseline: −2.30 ± 2.87.

a Nutritional status at baseline was available for 152 patients.

**Table 2:** Baseline characteristics according to telemedicine group.

Variable	No telemedicine	Telemedicine	*p*-value
Age (years)	9.06 ± 4.75	7.57 ± 4.61	0.056
PEG, *n* (%)	34 (38.2%)	38 (58.5%)	**0** **.** **013** *
GMFCS severe (IV-V), *n* (%)	52 (58.4%)	37 (56.9%)	0.852
Nutritional status at baseline, *n* (%)			0.430
Severe malnutrition, *n* (%)	28 (31.5%)	22 (34.9%)	
Moderate malnutrition, *n* (%)	16 (18.0%)	13 (20.6%)	
Normal, *n* (%)	32 (36.0%)	25 (39.7%)	
Diagnosis			**0**.035*
Encephalopathy (including cerebral palsy)	33 (37.1%)	21 (32.3%)	
Genetic syndromes	12 (13.5%)	19 (29.2%)	
Neurodegenerative diseases	16 (18.0%)	8 (12.3%)	
Neurodevelopmental disorders	1 (1.1%)	0 (0.0%)	
Neurometabolic diseases	6 (6.7%)	0 (0.0%)	
Neuromuscular diseases	21 (23.6%)	17 (26.2%)	

Baseline characteristics of patients according to telemedicine and standard care groups. Continuous variables are reported as mean ± standard deviation and compared using t-test. Categorical variables are expressed as counts and percentages and compared using chi-square or Fisher**’**s exact test when appropriate based on expected cell counts. GMFCS severe includes levels IV-V. Nutritional status was available for 152 patients.

* *p* < 0.05.

Nutritional outcomes according to telemedicine exposure are reported in Table 3. Follow-up data were available for 112 patients. The participant flow is summarized in Figure 1. Of the 152 patients included in the baseline analyses, follow-up data were unavailable for 40 patients at the time of data extraction because the study is ongoing and follow-up schedules reflect routine clinical practice. Baseline characteristics were compared between participants with and without follow-up data. No significant differences were observed in age (*p* = 0.909), baseline BMI z-score (*p* = 0.928), or follow-up modality (telemedicine vs standard care, *p* = 0.644). In addition, the proportion of patients with available follow—up data did not differ significantly between the telemedicine and standard care groups (74.2% vs 70.8%, *χ*^2^= 0.08, *p* = 0.777). However, patients with PEG were more likely to have available follow-up data than those without PEG (52.7% vs 31.0%, *p* = 0.015). The mean follow-up duration was 8.2 ± 5.7 months. No significant differences were observed between groups in BMI z-score at follow-up (−2.35 ± 2.66 vs −2.25 ± 4.05, *p* = 0.877) or in the change in BMI z-score (*p* = 0.458). The distribution of nutritional status at follow-up did not differ significantly between groups (*p* = 0.356). Similarly, no significant differences were observed in nutritional outcome categories (improved, stable, worsened) (*p* = 0.329). A higher proportion of patients in the telemedicine group experienced worsening of nutritional status compared to the standard care group (43.5% vs 30.3%); however, this difference did not reach statistical significance.

**Table 3:** Nutritional outcomes.

Variable	No telemedicine	Telemedicine	*p*-value
BMI z-score follow-up	−2.25 ± 4.05	−2.35 ± 2.66	0.877
Change in BMI z-score	−0.18 ± 2.93	0.20 ± 2.22	0.458
Nutritional status at follow-up, *n* (%)			0.356
Severe malnutrition	19 (28.8%)	15 (31.9%)	
Moderate malnutrition	12 (18.2%)	6 (12.8%)	
At risk of malnutrition^a^	7 (10.6%)	11 (23.4%)	
Normal	25 (37.9%)	14 (29.8%)	
Overweight	3 (4.5%)	1 (2.1%)	
Nutritional outcome, *n* (%)			0.329
Improved	34 (51.5%)	18 (39.1%)	
Stable	12 (18.2%)	8 (17.4%)	
Worsened	20 (30.3%)	20 (43.5%)	

Nutritional outcomes follow-up according to telemedicine and standard care groups. Continuous variables are presented as mean ± standard deviation and compared using t-test. Categorical variables are expressed as count and percentages and compared using chi-square tests.

a The “at risk” category was retained at follow-up for descriptive purposes.

In multivariable logistic regression analysis, baseline BMI z-score was significantly associated with nutritional worsening (OR 1.23, 95% CI 1.04-1.50; *p* = 0.013), with higher baseline BMI z-scores associated with higher odds of deterioration. No significant associations with nutritional worsening were observed for telemedicine exposure (*p* = 0.166), age (*p* = 0.767), PEG (*p* = 0.869), GMFCS severity (*p* = 0.704), or follow-up duration (*p* = 0.352) (Table 4).

**Table 4:** Predictors of nutritional deterioration (multivariable logistic regression).

Variable	OR (95% CI)	*p*-value
Telemedicine	0.74 (0.47 –1.18)	0.166
Age (years)	1.01 (0.92 –1.11)	0.767
PEG (yes)	0.96 (0.63 –1.46)	0.869
GMFCS severe (IV–V)	0.91 (0.56 –1.49)	0.704
BMI z-score baseline	1.23 (1.04 –1.50)	**0** **.** **013** *
Follow-up duration (months)	1.03 (0.96 –1.11)	0.352

Multivariable logistic regression analysis for factors associated with nutritional worsening. Odds ratios (OR) and 95% confidence intervals (CI) are reported. BMI z-score at baseline was included as a continuous variable. GMFCS severe includes levels IV-V.

* *p* < 0.05.

### 3.1 Sensitivity analysis using propensity score matching

A propensity score matching sensitivity analysis was performed to account for baseline differences between groups. After exclusion of one participant with missing baseline BMI z-score, 111 patients with complete data were available for the propensity score matching analysis. One-to-one nearest-neighbor matching yielded 36 matched pairs (*n* = 72). Baseline characteristics after matching are reported in Supplementary Table 2 and showed substantial improvement in covariate balance, with all standardized mean differences below 0.20, indicating acceptable balance across matched groups. Mean change in BMI z-score remained comparable between groups (telemedicine: +0.25 ± 2.22 vs standard care: −0.07 ± 3.22; *p* = 0.628) (Supplementary Table 2), confirming the robustness of the primary findings.

## 4 Discussion

In this longitudinal study, we evaluated the association between telemedicine follow-up and nutritional outcomes in children with neurological impairment. The main findings of this study are that malnutrition was highly prevalent in this population, and that baseline nutritional status was the primary factor associated with subsequent nutritional deterioration. No statistically significant differences in nutritional outcomes were observed between telemedicine and standard care groups.

Malnutrition is a well-recognized and frequent complication in children with neurological impairment, particularly in those with severe motor dysfunction and feeding difficulties. Previous studies have reported a high prevalence of undernutrition in this population, often exceeding 40-50%, depending on disease severity (15, 18, 20, 21, 41, 42). In our study, approximately half of the children presented with malnutrition at baseline, confirming the high nutritional vulnerability of this group. The observed association between malnutrition and PEG likely reflects the greater clinical severity of patients requiring enteral feeding, rather than a negative effect of PEG itself. Indeed, PEG is typically indicated in children with more severe feeding impairment and nutritional compromise and therefore represents a marker of disease severity rather than a protective factor (15, 17, 19, 20, 43).

Children in the telemedicine group presented with a more complex clinical profile at baseline. This was reflected by a higher prevalence of PEG and differences in the distribution of underlying diagnoses. The distribution of underlying diagnoses differed between groups, with a higher proportion of genetic syndromes in the telemedicine group, while encephalopathies remained the most represented category in both groups. Although GMFCS severity did not differ significantly between groups, these findings suggest that telemedicine was more frequently implemented in patients requiring closer monitoring or presenting with greater clinical needs. This observation is consistent with previous studies reporting that telemedicine is often used in patients with higher clinical complexity or barriers to accessing in-person care (2, 9, 44).

At follow-up, no statistically significant differences were observed between groups in BMI z-score, change in BMI z-score, or categorical nutritional outcomes. A higher proportion of patients in the telemedicine group experienced worsening of nutritional status compared to the standard care group, however, this difference was not statistically significant. The higher proportion of nutritional worsening observed in the telemedicine group should be interpreted in the context of baseline differences, as patients receiving telemedicine presented with greater clinical complexity. Overall, these findings suggest that short-term nutritional outcomes in this population are primarily driven by baseline nutritional status rather than by the modality of follow-up. Given the greater clinical complexity of patients in the telemedicine group at baseline, these findings should be interpreted with caution, as baseline differences between groups may have contributed to the observed trend. To further address this issue, a propensity score matching sensitivity analysis was performed using age, sex, PEG status, GMFCS severity, diagnostic category, and baseline BMI z-score. After matching, baseline covariate balance substantially improved, and the results remained unchanged, with no significant difference in BMI z-score change between groups. These findings support the robustness of the primary analysis and suggest that the observed results were not solely driven by baseline differences between groups. Telemedicine is increasingly used in the management of children with medical complexity and chronic conditions, particularly to support follow-up and improve access to care (12, 45). Previous studies have also highlighted high levels of caregiver satisfaction and acceptability of telehealth services (46), as well as its potential role in reshaping care delivery models (47). In our study, the absence of significant differences in nutritional outcomes persisted after propensity score matching, suggesting that telemedicine was not associated with either improved or worsened short-term nutritional trajectories. Within this context, telemedicine should be interpreted as a supportive tool for follow-up and continuity of care rather than as a direct determinant of clinical outcomes.

In the multivariable analysis, baseline BMI z-score was the only variable significantly associated with nutritional worsening. Interestingly, higher baseline BMI z-scores were associated with increased odds of deterioration during follow-up. This finding should be interpreted with caution, as nutritional outcome was defined according to changes in BMI z-score rather than absolute nutritional status. Children with higher baseline BMI values may have had greater opportunity to experience a decline exceeding the predefined threshold, whereas those with more severe malnutrition at baseline may have had less room for further deterioration or may have received more intensive nutritional interventions. In addition, this association may partly reflect a regression-to-the-mean phenomenon, whereby children with relatively higher baseline BMI z-scores may have been more likely to experience subsequent decreases over time. Because nutritional worsening was defined according to changes in BMI z-score rather than absolute nutritional status, the observed association may also reflect the mathematical coupling between baseline values and subsequent change. Therefore, this finding should be interpreted cautiously. In contrast, GMFCS severity and PEG, although associated with malnutrition at baseline, were not independently associated with worsening in the adjusted model. Given the observational design and limited sample size, this finding should be considered exploratory and warrants further investigation in larger prospective studies. This finding underscores the importance of early identification and management of malnutrition in children with neurological impairment (48).

This study has several limitations. First, the observational design and the lack of randomization in the allocation to telemedicine follow-up introduce potential selection bias. Although a propensity score matching sensitivity analysis yielded findings consistent with the primary analysis, residual confounding due to unmeasured variables cannot be excluded. Second, the telemedicine group included patients with greater clinical complexity at baseline, limiting comparability between groups and precluding causal inference regarding the effect of telemedicine on nutritional outcomes. The results remained consistent with the primary analysis, supporting the robustness of the study findings despite baseline differences between groups. Third, anthropometric data collected during telemedicine follow-up were partially based on caregiver-reported measurements, which may be subject to variability. Although caregivers received standardized verbal instruction during in-person visits regarding weight and length measurements, measurement error cannot be completely excluded and may have affected the accuracy of some anthropometric assessments. In addition, BMI z-score, although widely used in pediatric nutritional assessment, may not fully capture nutritional status in children with severe neurological impairment because of altered body composition, reduced muscle mass, and condition-specific growth patterns. Therefore, BMI should be interpreted as a pragmatic indicator of nutritional trajectory rather than a comprehensive measure of nutritional status in this population. Follow-up intervals were not standardized and reflect routine clinical practice. In addition, follow-up data were not available for a proportion of participants at the time of data extraction, which may have introduced attrition bias. However, patients with and without follow-up data did not differ significantly in age, baseline BMI z-score, or follow-up modality, although a higher prevalence of PEG was observed among participants who completed follow-up. Because the study is ongoing and follow-up schedules reflected routine clinical practice, some participants had multiple follow-up visits whereas others had only one. To ensure comparability across patients, analyses were restricted to the first available follow-up measurement after baseline. Consequently, longer-term nutritional trajectories and within-subject changes across multiple assessments could not be evaluated. Although longitudinal approaches such as mixed-effects models may be appropriate when repeated measurements are available across multiple time points, the heterogeneous number of follow-up assessments and the limited number of participants with repeated observations precluded a robust longitudinal modelling approach in the present study.

Despite these limitations, this study provides clinically relevant insights into the use of telemedicine in the nutritional management of children with neurological impairment. Telemedicine can be considered a feasible approach to support follow-up, facilitate monitoring, and provide dietary counselling, particularly in patients requiring frequent assessment or facing barriers to in-person care (1). However, despite adjustment for baseline differences through multivariable analysis and propensity score matching, the observational design of the study precludes causal inference regarding the effect of telemedicine on clinical outcomes.

## 5 Conclusion

Baseline BMI z-score was the only variable associated with nutritional worsening during follow-up. In this study, no statistically significant differences in nutritional outcomes were observed between telemedicine and standard care groups; however, differences in baseline characteristics limit direct comparability between groups. Telemedicine may represent a feasible and complementary approach to support nutritional monitoring and continuity of care, particularly for children requiring frequent follow-up or facing barriers to in-person visits. However, these findings should be interpreted in light of the observational design and baseline group differences, and do not allow causal inferences regarding the relationship between telemedicine and clinical outcomes. Further prospective studies are needed to better define its role in long-term nutritional management and to evaluate its impact on clinical outcomes in larger and more homogeneous populations.

## Data Availability

The raw data supporting the conclusions of this article will be made available by the authors, without undue reservation.
