# Supplementary material for: Telemedicine follow-up and nutritional outcomes in children with neurological impairment: a longitudinal study
Source: Front Pediatr. 2026 Jul 8;14:1868448. doi: 10.3389/fped.2026.1868448 (PMC13388881; doi:10.3389/fped.2026.1868448)
Supplement: Supplementary file 1 [file Table1.docx]

| **Supplementary table 1 - Characteristics of the study population** |  |
| --- | --- |
| **Variable** | **Total sample** |
| Age (years) | 8.52 ± 4.85 |
| Sex (M/F) | 97 (63%) / 57 (37%) |
| PEG (Y/N) | 72 (46.7%) / 82 (53.2%) |
| BMI z-score baseline | -2.30 ± 2.86 |
| Follow up duration (months) | 8.24 ± 5.67 |
| **GMFCS** |  |
| GMFCS I | 3 (1.9%) |
| GMFCS II | 3 (1.9%) |
| GMFCS IV | 5 (3.2%) |
| GMFCS V FO | 10 (6.4%) |
| GMFCS V NT | 30 (18.6%) |
| GMFCS V TF | 44 (28.5%) |
| **Growth charts** |  |
| Bertoli et al. | 1 (0.6%) |
| WHO | 8 (5.1%) |
| CDC | 26 (16.8%) |
| **Disease specific-charts** |  |
| SMA I | 19 (12.3%) |
| SMA II | 3 (1.9%) |
| PWS | 1 (0.6%) |
| Rett syndrome | 1 (0.6%) |
| **Nutritional status baseline** |  |
| Normal | 42 (27.2%) |
| Moderate malnutrition | 29 (18.8%) |
| Severe malnutrition | 50 (32.4%) |
| At risk of malnutrition | 26 (16.8%) |
| Overweight | 4 (2.5%) |
| Obesity | 3 (1.9%) |
| **Diagnosis group** |  |
| - Encephalopathy (including Cerebral Palsy) | 54 (35.1%) |
| - Genetic syndromes | 31 (20%) |
| - Neuromuscular diseases | 38 (24.6%) |
| - Neurodegenerative diseases | 24 (15.5%) |
| - Neurodevelopmental disorders | 1 (0.6%) |
| - Neurometabolic diseases | 6 (3.8%) |
| **Group** |  |
| In-person visits | 89 (57.8%) |
| In-person visits + TLM | 65 (42.2%) |
|  |  |

Table 1: Baseline demographic and clinical characteristics of children with neurological impairment included in the study
