# Supplementary material for: Telemedicine follow-up and nutritional outcomes in children with neurological impairment: a longitudinal study
Source: Front Pediatr. 2026 Jul 8;14:1868448. doi: 10.3389/fped.2026.1868448 (PMC13388881; doi:10.3389/fped.2026.1868448)
Supplement: Supplementary file 2 [file Table2.docx]

**Supplementary Table 2. Baseline characteristics and nutritional outcomes after propensity score matching**

| **Variable** | **Standard care (n=36)** | **Telemedicine (n=36)** |
| --- | --- | --- |
| Age (years) | 9.00 ± 4.52 | 8.22 ± 4.66 |
| Male sex, n% | 23 (63.9%) | 23 (63.9%) |
| PEG n% | 21 (58.3%) | 21 (58.3%) |
| GMFCS severe (IV-V), n (%) | 25 (69.4%) | 23 (63.9%) |
| Baseline BMI z-score | -2.45 ± 2.74 | -2.18 ± 3.09 |
| **Diagnosis, n (%)** |  |  |
| Cerebral Palsy | 5 (13.9%) | 4 (11.1%) |
| Encephalopathies | 10 (27.8%) | 10 (27.8%) |
| Genetic syndromes | 7 (19.4%) | 6 (16.7%) |
| Neurodegenerative diseases | 6 (16.7%) | 6 (16.7%) |
| Neurometabolic diseases | 0 (0.0%) | 0 (0.0%) |
| Neuromuscular disorders | 8 (22.2%) | 10 (27.8%) |
| **Nutritional outcome after matching** |  |  |
| Δ BMI z-score | -0.07 ± 3.22 | 0.25 ± 2.22 |

**Supplementary Table 2**. Baseline characteristics and nutritional outcomes after 1:1 propensity score matching. propensity scores were estimated using age, sex, PEG status, GMFCS severity, diagnostic category, and baseline BMI z-score. One-to-one nearest-neighbor matching without replacement (caliper=0.2) yielded 36 matched pairs. Continuous variables are presented as mean ± standard deviation and categorical variables as counts and percentages.
